# Comparative susceptibility of various pulses and their impact on the biological traits of pulse beetle, *Callosobruchus chinensis* (Linn.) (Coleoptera: Chrysomelidae)

**DOI:** 10.1038/s41598-026-46013-y

**Published:** 2026-03-30

**Authors:** Vasu Mehta, R S Chandel, Jayaram CS

**Affiliations:** 1 Department of Entomology, CSK Himachal Pradesh Agricultural University, 176062 Palampur, Himachal Pradesh India; 2 Department of Entomology, Faculty of Agriculture, Guru Kashi University, Talwandi Sabo, 151302 Bathinda, Punjab India; 3 AGRIHAWK Technologies PVT LTD, 560102 Bengaluru, Karnataka India

**Keywords:** Pulse beetle, Biology, Pulses, Damage, Germination, Weight loss, Orientation, Developmental biology, Zoology

## Abstract

Pulses play a significant role in Indian agriculture, contributing notably to the country’s economy through substantial export earnings. As the world’s leading producer of pulses, India benefits greatly from their cultivation. Pulses are rich in protein, containing 20 to 25 percent by weight, which is twice the protein content found in wheat and three times that of rice. Pulses are infested by many insect-pests during storage, however, pulse beetle, *Callosobruchus chinensis* is one of the major pest causing substantial losses in different pulses. Out of eight pulses investigated, pulse beetle completed its life cycle on all the pulses with varying time periods. Highest susceptibility index and adult emergence was found in case of green gram while lowest was in case of kidney bean. Talking about the damage and weight loss, it was maximum in case of green gram followed by chickpea while lowest was in case of horse gram and kidney bean. Pulse beetle also affected the germination potential of seeds of every pulse evaluated, however green gram, chickpea, kabuli chickpea and soybean were the most affected ones. Overall, green gram was ranked as most susceptible pulse while, kidney bean followed by horse gram was ranked as least susceptible pulse against *C. chinensis*. Therefore, concluding the study, green gram and chickpea were found to be most preferred host for *C. chinensis* and kidney bean and horse gram were least preferred hosts when compared on the basis of parameters like biology, grain damage, weight loss and germination.

## Introduction

Bruchids, also known as pulse beetles, pea beetles, or bean beetles, primarily feed on legumes of the Phaseoleae tribe^1^. The genus *Callosobruchus* is widespread across tropical and subtropical regions, infesting seeds of mung beans, chickpeas, and other pulses both before and after harvest. In India, 117 bruchid species from 11 genera have been identified as pests of various pulses^2^, with *C. maculatus*, *C. analis*, and *C. chinensis* being the most common^3^. While the beetles cause minimal damage in the field, stored infested seeds are more vulnerable. Adults emerge from the seeds and lay eggs on nearby seeds, leading to secondary infestations that are far more destructive. Female bruchids lay eggs individually on the grain sheath in the field or on dried seeds during storage. The larvae hatch and burrow into the seed beneath the egg, with the egg shell adhering to the seed^4,5^. The larvae remain inside the seed, and the presence of a capped exit hole indicates the pupal stage^5^. The adults do not require food or water and can reproduce immediately after emerging^6,7^. The pest’s entire immature life cycle occurs within a single seed, resulting in weight loss, reduced germination potential, and a decline in both the market and nutritional value of the affected legumes^8^.

Understanding the distribution and biology of insect pests is crucial for effectively managing stored grain pests^9^. This knowledge helps identify vulnerable life stages and track the pest population’s growth^10^. Temperature, which is influenced by environmental conditions, plays a key role in the population dynamics and timing of insect biological processes^11^. Estimating storage losses at the farm level has long been a challenging issue. While numerous surveys and estimates have been conducted over time, the exact losses caused by various factors in rural areas remain unclear. The development, population increase, and level of infestation caused by insect pests are influenced by the type of food available, but the relationship between food and insect development has not been thoroughly explored.

Pulses are a crucial crop in many regions, including Himachal Pradesh, where they are often stored in both modern bins and traditional storage methods for personal consumption and as seed stock. These crops face significant damage from various insect pests, with the pulse beetle being one of the most destructive, causing substantial losses at both the farm and storage levels^8,12,13^. To manage this pest effectively, it is essential to understand the beetle’s biology in relation to different pulse crops and assess the full extent of the damage it causes, both in terms of quality and quantity. This study was conducted to address these important issues.

## Material and methods

The present investigation was conducted under laboratory conditions in the Economic Entomology laboratory of Department of Entomology, College of Agriculture, CSK Himachal Pradesh Agricultural University, Palampur, Himachal Pradesh, India (32.0986° N, 76.5430° E, 1250 m amsl) during 2023–24. The detail of the treatments, materials used, techniques followed and criteria adopted for various experiments during the course of present investigations are described in the following text.

### Maintenance of stock culture of pulse beetle

**Fig. 1:**
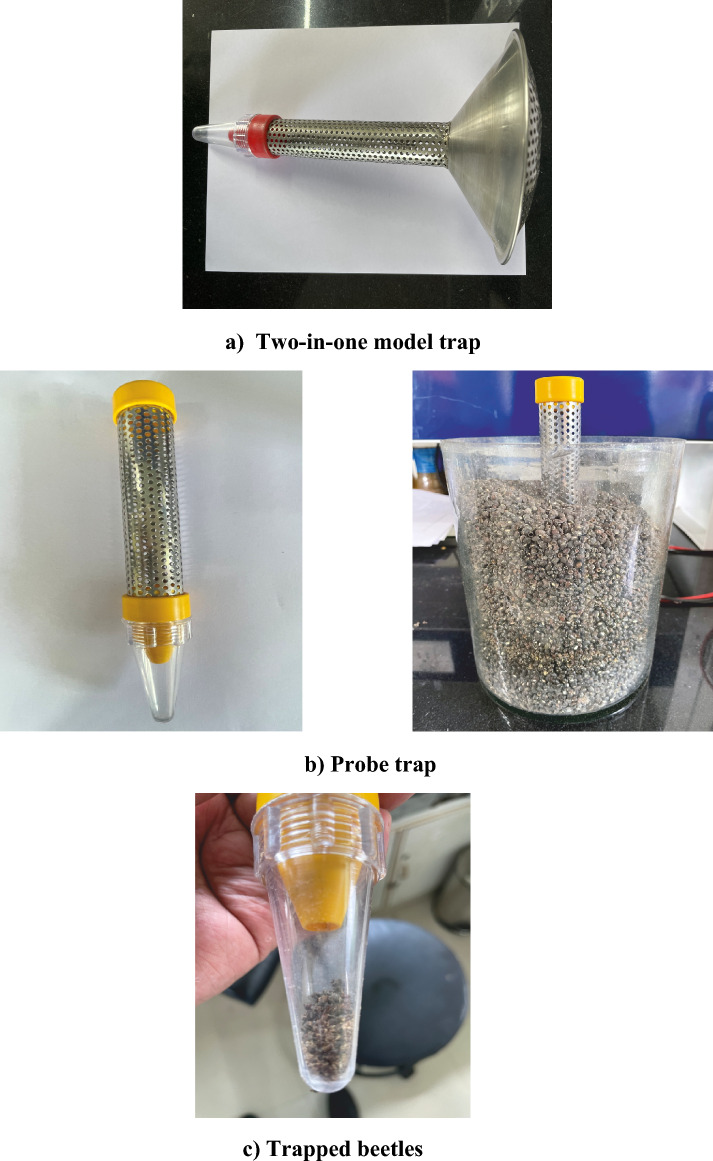
Collection of adults of *Callosobruchus chinensis*.

Stock culture of pulse beetle was maintained on green gram, black gram, chickpea and kidney bean and initiated by collecting the adult beetles from the infested grains from farmer’s storage containers and local market using ‘Two-in-one pitfall trap’ and ‘probe tap’ (Fig. 1). To maintain pulse grains in healthy condition, they were cleaned and sieved to remove the fractions of unhealthy grains or insects, if any. The grains were disinfected in order to eliminate both hidden and apparent infestation, if any. The culture was further multiplied and maintained in the laboratory (26 ± 2^0^C, 14L: 10D and 70% RH). Fresh pulse seeds were provided ad libitum periodically for the development of the beetle. Grains with eggs from the stock culture were separated and placed in a new large plastic jar (14 cm x 14 cm x 17 cm) to get pure culture of pulse beetle. The culture so maintained and newly emerging beetles were used to maintain the pure culture of *C. chinensis*.

### Pure culture of pulse beetle

**Fig. 2:**
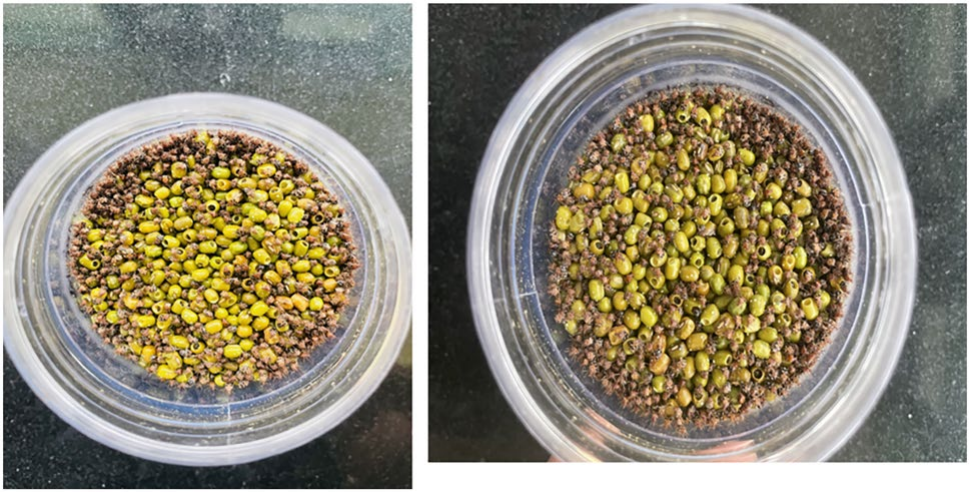
Pure culture of *Callosobruchus chinensis*.

The pure culture of predominant species of pulse beetle, *C. chinensis* was maintained on green gram in plastic jars of 2 kg capacity (14 cm x 14 cm x 17 cm). The maintained culture was purely of *C. chinensis* as it was identified by two institutes i.e. CIPHET, Ludhiana, Punjab, India and CSIR-IHBT, Palampur, Himachal Pradesh. The open tops of the jar were covered with clean sterile muslin cloth and properly tightened with rubber bands (Fig. 2). Newly emerged adults from stock culture were released in each jar containing one and half kg of green gram seeds. The jars were labeled and placed in the laboratory (26 ± 2^0^C, 14L: 10D and 70% RH).. After allowing the beetles to oviposit for more than one week, these were removed from the jars using ‘Two-in-one pitfall trap’ and ‘probe tap’ and were discarded. The newly emerged adults were used for further experimentation and some of them were transferred to fresh grains. Fresh culture on new green gram seeds was raised time to time from newly emerged pulse beetles to avoid any type of fungal infection and buildup of any type of parasite.

### Collection of different pulses

The seeds of eight different pulses viz*.,* green gram (*Vigna radiata* L.) variety DPM 8909, black grams (*Vigna mungo* (L.)) variety Him Mash-1, soybeans **(***Glycine max* (L.) Merr.) variety Himso-1685, cowpeas (*Vigna unguiculata* (L.) Walp.) variety CL 367, chickpeas (*Cicer arietinum* L.) variety Himachal Chana 2, kabuli chickpeas (*Cicer kabulium* L.) variety HK-1, lentils (*Lens culinaris* Medik.) variety Vipasha, kidney beans (*Phaseolus vulgaris* L,) variety Jwala were obtained from Department of Seed Science and Technology, CSK Himachal Pradesh Agricultural University, Palampur, Himachal Pradesh (Table 1). The seeds were healthy, genetically pure, insect-free, and disease-free. The seeds were further critically examined and foreign material was removed from the lot. The seeds were stored in plastic containers and appropriately covered with sterile, clean muslin cloth for future testing. The seeds were further critically examined and foreign material was removed from the lot. The seeds were stored in separate plastic containers (14 cm x 14 cm x 17 cm), properly covered with clean sterile muslin cloth for future testing.

**Table 1:** Different pulses screened against *Callosobruchus chinensis*.

S. no	Pulses	Scientific name	Weight of 100 seeds (g)
1	Cowpea	*Vigna unguiculata*	21.024
2	Chickpea	*Cicer arietinum*	22.643
3	Horse gram	*Macrotylom auniflorum*	3.090
4	Kabuli chickpea	*Cicer kabulium*	54.701
5	Kidney bean	*Phaseolus vulgaris*	35.562
6	Black gram	*Vigna mungo*	4.709
7	Green gram	*Vigna radiata*	3.439
8	Soybean	*Glycine max*	26.228

### Biological parameters of pulse beetle

The biology of *Callosobruchus chinensis* (Linn.) was studied under laboratory conditions (26 ± 2^0^C, 14L: 10D and 70% RH) on different pulses viz., green gram, black gram, soybean, cowpea, chickpea, kabuli chickpea, lentil and kidney bean. Each pulse @100 g was stored in plastic box of 100 g capacity (5.3 cm × 5.3 cm x 7 cm) and a total of 10 pairs (20 adults) of *C. chinensis* were released in each box. The experiment was replicated thrice and observations were recorded on different biological parameters like incubation period, larval period, pupal period, orientation, fecundity, developmental period, adult longevity and adult emergence. The grains were examined daily and regular counts were made on newly emerged adults to find out the total adult emergence. Total developmental period of the beetle was recorded as the total number of days taken by the beetle for completing its life cycle from oviposition to adult emergence on different pulses.

The susceptibility index of each pulse was calculated by applying the formula suggested by Dobie^14^ as given below:add space between Susceptibility index in the formula.

$$\mathbf{S}\mathbf{u}\mathbf{s}\mathbf{c}\mathbf{e}\mathbf{p}\mathbf{t}\mathbf{i}\mathbf{b}\mathbf{i}\mathbf{l}\mathbf{i}\mathbf{t}\mathbf{y}\:\:\mathbf{i}\mathbf{n}\mathbf{d}\mathbf{e}\mathbf{x} =\frac{\text{Natural log F x }100}{D}$$

where,

$$\text{F }=\text{ Number of adults emerged}$$

$$\text{D }=\text{ Mean developmental period}$$

### Orientation of C. chinensis towards different pulses

For orientation studies, 20 g of each pulse was kept in small square boxes (15.24 cm × 12.7 cm × 3.18 cm) which were further placed in a large square plastic box (22 cm x 22 cm × 6.5 cm). One hundred adults of *C. chinensis* were released in the center of the trough, and minute holes were made with sharp pin on the box lid for proper aeration. The experiment was replicated thrice and the number of adults oriented towards each pulse was counted at 24, 48 and 72 h of initial release.

### Estimation of losses caused by pulse beetle in different pulses

The healthy grains of different pulses viz. soybean, chickpea, horse gram, kidney bean, cowpea and black gram were thoroughly examined in order to eliminate previous infestation of stored grain pests. Grains of each pulse (100 g) were kept in plastic box of 100 g capacity (5.3 cm × 5.3 cm x 7 cm). Ten pairs of about five-day old adults of predominant specie of pulse beetle i.e. *C. chinensis* were released in each container having proper aeration. These were kept in three replications, and observations on weight loss of grains and % infestation was recorded after 30, 60, 80, 100 and 120 days. The data will be subjected to statistical analysis after appropriate transformation. The losses were estimated using the formula given by Adams and Schulten^15^:

- $$\text{Grain damage }(\mathrm{\%}) =\text{ no}.\text{ of grains damaged}/\text{total no}.\text{ of grains in a vial x}100$$
- $$\text{Weight loss }(\mathrm{\%}) =\frac{(\mathrm{UND}) - (\mathrm{DNU})\text{ x }100}{\text{U }(\mathrm{ND}+\mathrm{NU})}$$
- U—wt. of un-damaged grains (g)
- NU— no. of un-damaged grains
- D—wt. of damaged grains
- ND—no. of damaged grains

### Germination test

For evaluating germination potential of seeds of different pulses after being damaged, germination test from 50 randomly selected seeds was carried out after 80 and 120 days of storage. Paper towel method of germination test was employed following the standard methodology given by International Seed Testing Association (ISTA). The seeds were placed in germinator at 25° C, with 85 per cent relative humidity. Germination counts were taken on 7^th^ day after incubation. The per cent loss in germination was worked out, replication-wise for each treatment and was calculated by the formula:add space between Per cent germination in the formula

$$\mathbf{P}\mathbf{e}\mathbf{r}\:\:\mathbf{c}\mathbf{e}\mathbf{n}\mathbf{t}\:\:\mathbf{g}\mathbf{e}\mathbf{r}\mathbf{m}\mathbf{i}\mathbf{n}\mathbf{a}\mathbf{t}\mathbf{i}\mathbf{o}\mathbf{n}=\frac{\text{Number of grains germinated}}{\text{Total number of grains used}}\mathrm{x}100$$

### Ranking of different pulses based on their susceptibility

**Fig. 3:**
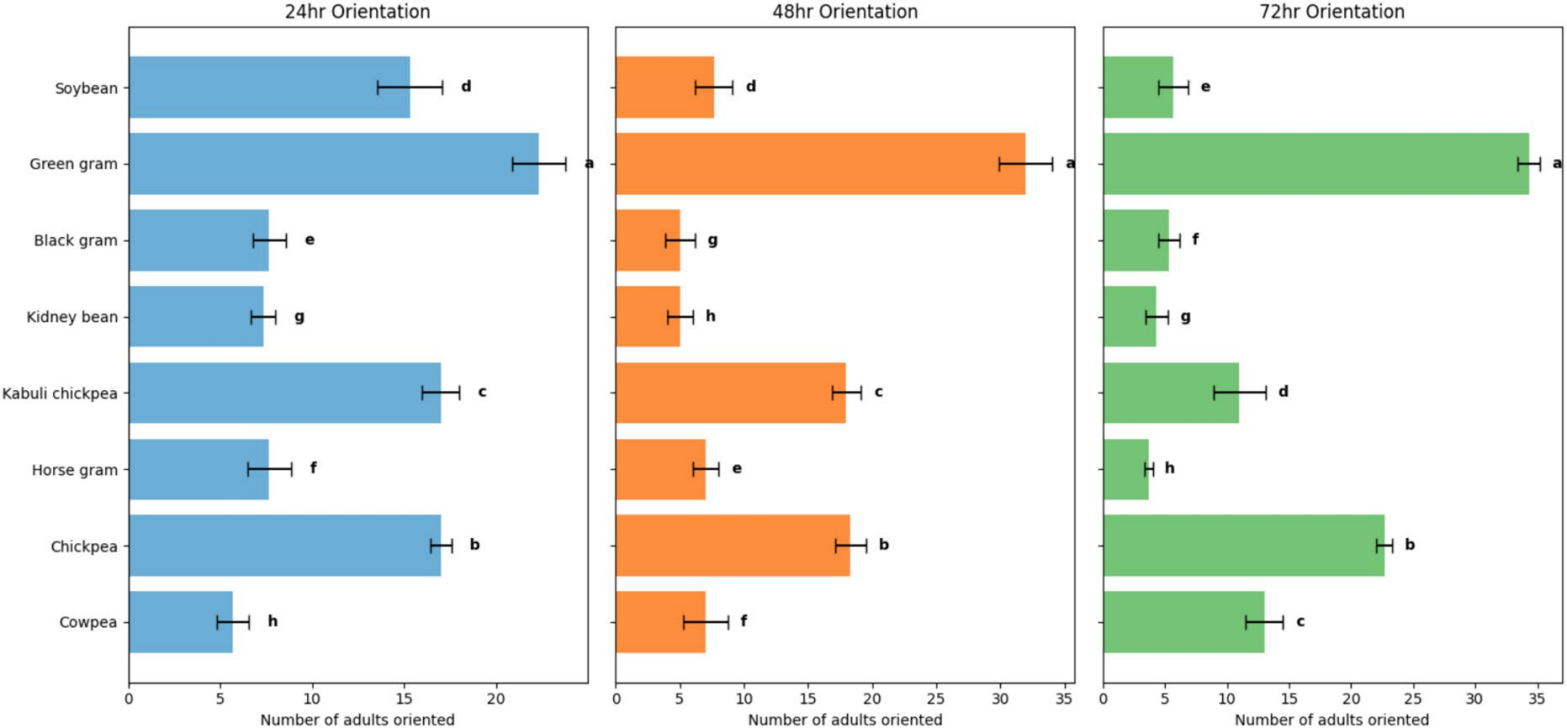
Orientation of *Callosobruchus chinensis* towards different pulses.

Different pulses were ranked (Rank 1–8) based on the biological parameters and losses caused by pulse beetle according to the methodology followed by^16^. The least susceptible pulse was ranked as 1, while the most susceptible was ranked as 8. At last, ranks obtained from different parameters were combined to get a final rank of each pulse (Table 7,Fig. 3).

### Statistical analysis

The biological parameters were subjected to analysis of variance (ANOVA) using OPSTAT and WASP 2.0 software. Means were separated using least significant difference (LSD) test at 5% probability level to determine significant differences among treatments. Descriptive statistics (mean ± standard error) were used for developmental durations and compared for each treatment. In case of all other experiments, the data obtained were subjected to statistical analysis in a completely randomized design with SPSS version 13.0 (IBM Corporation, Armonk, NY) for one-way analysis of variance. The significance of treatments was evaluated by Tukey’s honestly significant difference (HSD) test at P < 0.05 after being subjected to the appropriate transformations.

## Results

### Biology of C. chinensis

Pulse beetle deposited variable number of eggs on the surface of different pulses with highest number of eggs being laid on green gram (87.00) and lowest on kidney bean (56.33) while the range was mediocre in rest of the pulses (Table 2). The eggs of *C. chinensis* were cigar shaped and shiny bright yellow which turned slightly whitish before hatching. The incubation period varied from 3.33 days to 7.33 days among different pulses. It was shortest on green gram and longest on black gram while it ranged from 3.67 days to 6.67 among other pulses. Talking about the larva, they were fleshy, curved, creamy white in colour with black mouth parts and larval period was longest in case of black gram (21.67 days) followed by horse gram (21.00 days) and shortest in case of green gram (14.00 days) followed by chick pea (15.67 days). Pupa was exarate and creamy white and pupal period was longest in case of kidney bean (8.33 days) followed by horse gram (8.00 days) and it followed the same trend as larval period in case of shortest period i.e. green gram (4.67 days) followed by chickpea (5.33 days).

**Table 2:** Biology of *Callosobruchus chinensis* on different pulses.

Pulses	Fecundity	Incubation period	Larval period	Pupal period	Developmental period	Male longevity	Female longevity	Adult emergence	Susceptibility index
Cowpea	71.33 ± 1.45	4.33 ± 0.33	18.67 ± 1.20	7.33 ± 0.33	30.33 ± 0.88	6.67 ± 0.33	8.33 ± 0.33	79.00 ± 2.89	14.42 ± 0.39
Chickpea	83.33 ± 2.60	3.67 ± 0.33	15.67 ± 1.20	5.33 ± 0.33	24.67 ± 0.88	8.67 ± 0.33	11.00 ± 0.57	95.33 ± 1.45	18.51 ± 0.62
Horse gram	64.33 ± 1.76	5.33 ± 0.33	21.00 ± 1.15	8.00 ± 0.58	34.33 ± 0.88	6.00 ± 0.58	7.33 ± 0.33	61.33 ± 3.18	11.99 ± 0.24
Kabuli chickpea	79.33 ± 1.76	4.67 ± 0.67	16.00 ± 0.58	6.00 ± 0.58	27.00 ± 1.00	7.33 ± 0.33	9.67 ± 0.33	82.67 ± 2.19	16.39 ± 0.65
Kidney bean	56.33 ± 1.45	6.67 ± 0.33	18.67 ± 0.88	8.33 ± 0.33	33.67 ± 0.88	5.33 ± 0.33	7.00 ± 0.57	54.67 ± 1.45	11.89 ± 0.24
Black gram	61.33 ± 2.03	7.33 ± 0.33	21.67 ± 0.67	6.00 ± 0.58	35.00 ± 1.00	7.00 ± 0.58	8.00 ± 0.57	65.00 ± 2.30	11.94 ± 0.35
Green gram	87.00 ± 2.08	3.33 ± 0.33	14.00 ± 0.58	4.67 ± 0.33	22.00 ± 0.58	9.33 ± 0.33	12.33 ± 0.33	102.67 ± 3.71	21.08 ± 0.71
Soybean	74.00 ± 3.21	4.67 ± 0.33	17.00 ± 0.58	6.33 ± 0.33	28.00 ± 1.16	6.00 ± 0.58	7.67 ± 0.33	82.33 ± 4.06	15.78 ± 0.48
LSD (p ≤ 0.05)	**6.414**	**1.182**	**2.714**	**1.333**	**2.783**	**1.333**	**1.333**	**8.486**	**1.478**
SE(m)	2.121	0.391	0.898	0.441	0.920	0.441	0.441	2.806	0.489
SE(d)	3.000	0.553	1.269	0.624	1.302	0.624	0.624	3.969	0.691

Data on total developmental period of *C. chinensis* on different pulses varied significantly from 22 to 35 days. Maximum developmental period was observed in black gram while shortest was in case of green gram and rest of the pulses had mediocre periods. Perusal of data presented in Table 2 also indicates that adult emergence varied significantly from 54.67 to 102.67 adults among eight pulses. Maximum number of adults emerged from green gram which was followed by chickpea and kabuli chickpea and significantly lowest number of adults emerged in case of kidney bean. Male and female survived from 5.33 days to 9.33 days and 7.00 days to 12.33 days respectively on different pulses. Male and female longevity was highest in case of green gram and was lowest in case of kidney bean. Susceptibility index of different pulses was calculated on the basis of number of adults emerged and total developmental period of the beetle. Its value varied from 11.89 to 21.08 among different pulses. The highest value of susceptibility index was computed for green gram and least value of susceptibility index was calculated in case of kidney which was not significantly different from horse gram and black gram.

### Orientation studies

**Fig. 4:**
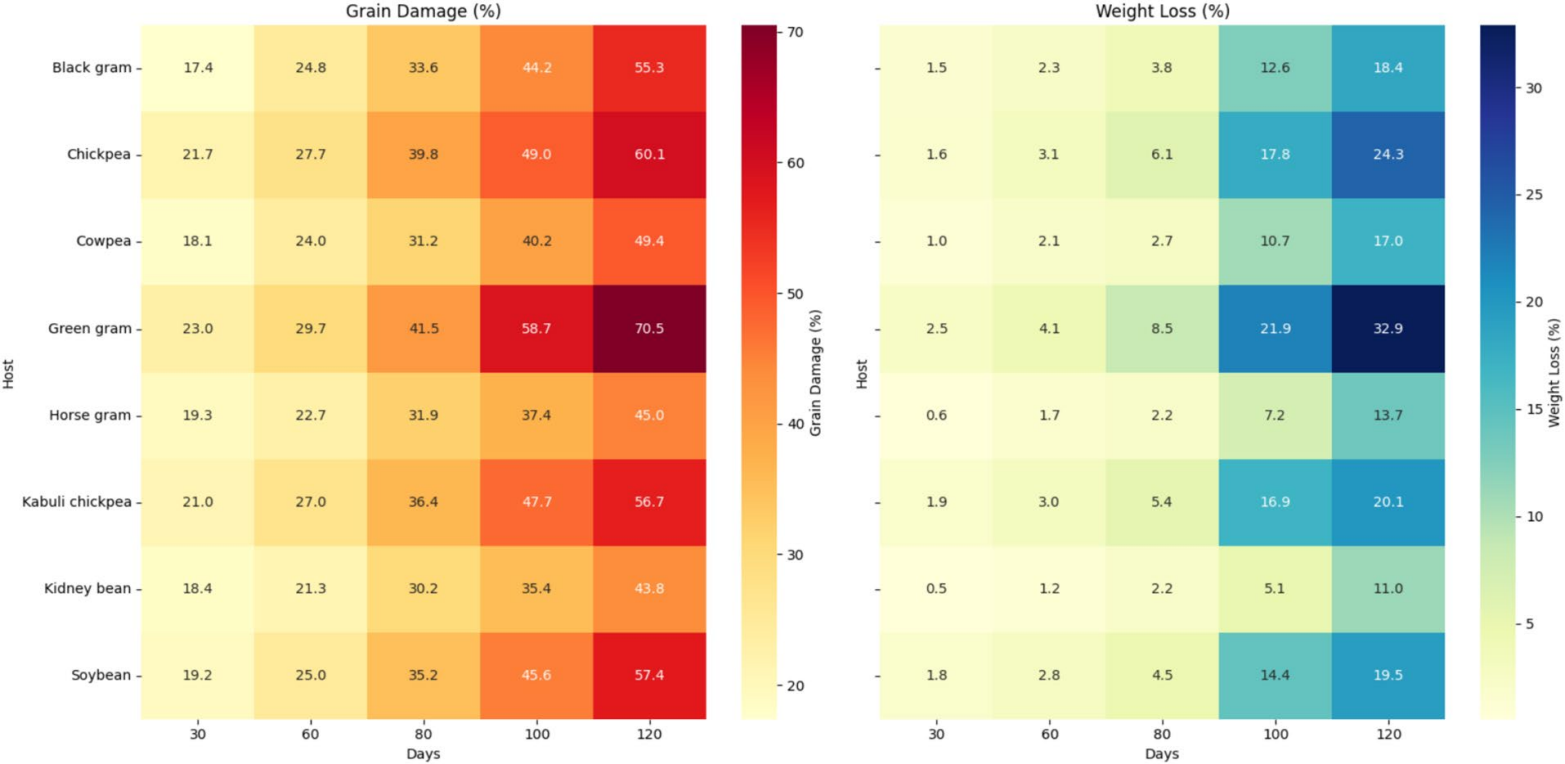
Quantitative losses caused by *Callosobruchus chinensis* in different pulses.

A separate experiment was conducted to study the orientation of *C. chinensis* adults towards different pulses after 24, 48 and 72 h of release (Table 3; Fig. 4). Data of the experiment revealed that, after 24 h of release, maximum orientation of adults was recorded towards green gram (22.33) followed by chick pea (17.00) and kabuli chick pea (17.00) and minimum orientation was recorded towards kidney bean (7.33) followed by horse gram (7.67) and black gram (7.67). After 48 and 72 h of release of beetles, maximum adult orientation followed the similar trend i.e. towards green gram while least adults oriented towards kidney bean and horse gram, respectively. The differences in minimum and maximum number of adults oriented towards different pulses after 24, 48 and 72 h of release were highly significant. Thus, the orientation preference after 72 h of release was in following decreasing order: green gram > chickpea > cowpea > kabuli chickpea > soybean > black gram > kidney bean > horse gram.

**Table 3:** Orientation of *Callosobruchus chinensis* towards different pulses.

S. No	Pulses	Number of adults oriented after		
		**24 h**	**48 h**	**72 h**
1	**Cowpea**	5.67^c^ ± 0.88	7^c^ ± 1.73	13^c^ ± 1.52
2	**Chickpea**	17^ab^ ± 0.57	18.33^b^ ± 1.20	22.67^b^ ± 0.66
3	**Horse gram**	7.67^c^ ± 1.20	7^c^ ± 1.00	3.67^e^ ± 0.33
4	**Kabuli chickpea**	17^ab^ ± 1.00	18^b^ ± 1.15	11^ cd^ ± 2.08
5	**Kidney bean**	7.33^c^ ± 0.66	5^c^ ± 1.00	4.33^e^ ± 0.88
6	**Black gram**	7.67^c^ ± 0.88	5^c^ ± 1.15	5.33^de^ ± 0.88
7	**Green gram**	22.33^a^ ± 1.45	32^a^ ± 2.08	34.33^a^ ± 0.88
8	**Soybean**	15.33^b^ ± 1.76	7.67^c^ ± 1.45	5.67^de^ ± 1.20
	**LSD (p ≤ 0.05)**	**(5.47)**	**(6.83)**	**(5.74)**

Values represent mean of three replicates; ± values are SE; means Followed by the same letter do not differ significantly at P = 0.05 according to Tukey’s HSD test.

### Grain damage caused by pulse beetle

**Fig. 5:**
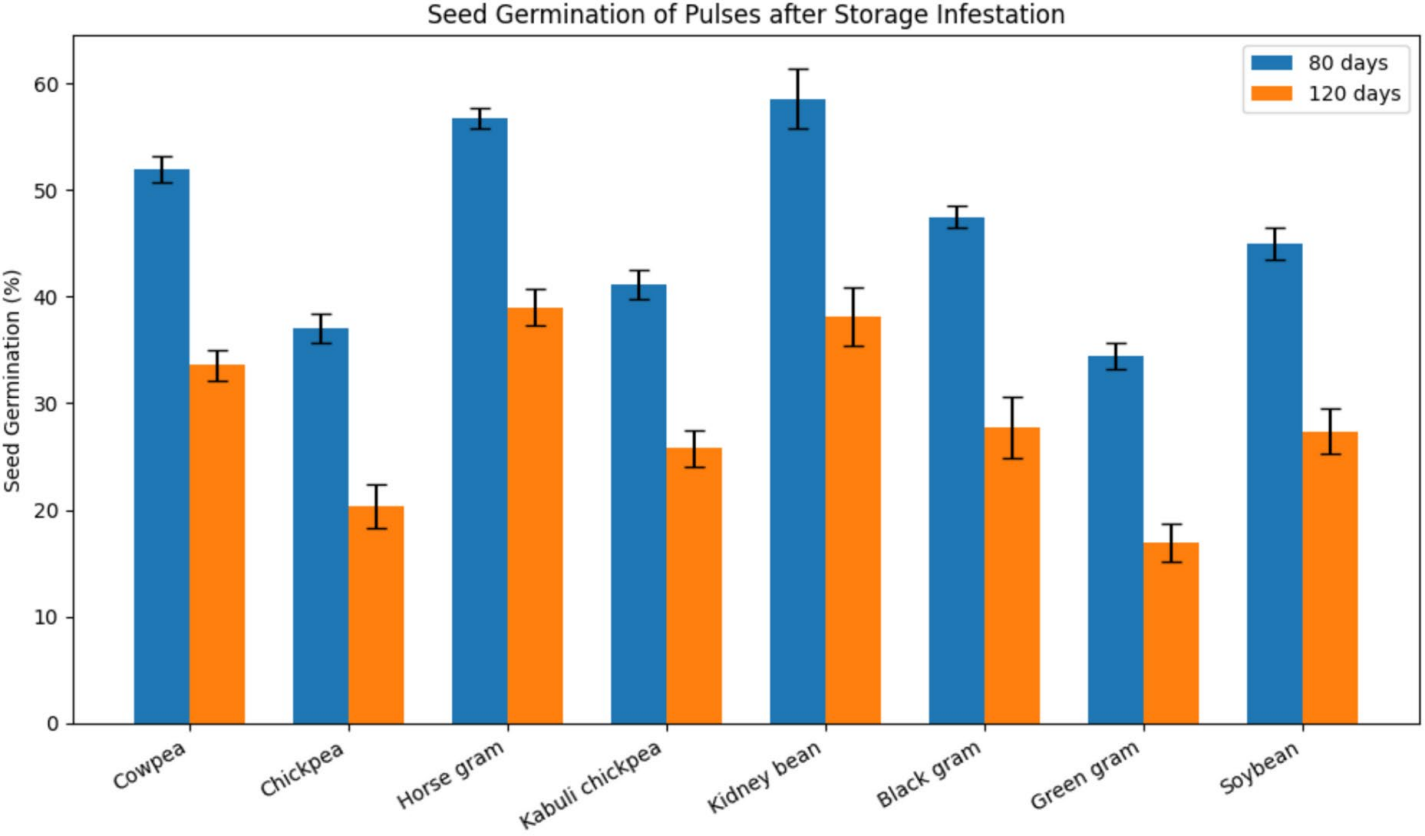
Effect of damage by *Callosobruchus chinensis* on germination of different pulses.

Grain damage caused by *C. chinensis* increased progressively with storage duration across all pulse hosts (Table 4; Fig. 5). At 30 days after beetle release, damage ranged from 17.38 percent (black gram) to 23.04 percent (green gram). By 120 days, damage had escalated substantially, reaching its highest level in green gram (70.49%), followed by chickpea (60.05%) and soybean (57.42%). Kabuli chickpea (56.67%) and black gram (55.33%) also suffered significantly high damage, while kidney bean (43.83%) recorded the lowest levels, comparable to horse gram (44.98%). Statistical analysis (LSD, p ≤ 0.05) confirmed that green gram consistently sustained the highest damage across all intervals, significantly differing from most other hosts. These findings highlight the relative susceptibility of green gram, chickpea, and soybean to bruchid infestation under storage conditions, whereas kidney bean and horse gram exhibited comparatively greater resistance.

**Table 4:** Damage potential of *Callosobruchus chinensis* on different pulses at Palampur.

Hosts	Grain damage after indicated days of weevil release (%)				
	**30**	**60**	**80**	**100**	**120**
**Cowpea**	18.088^ab^ ± 0.63	24.045^abc^ ± 1.12	31.243^c^ ± 1.69	40.179^cde^ ± 1.08	49.4^ cd^ ± 1.30
**Chickpea**	21.651^ab^ ± 0.99	27.705^ab^ ± 1.01	39.796^ab^ ± 0.67	49.024^b^ ± 2.00	60.054^b^ ± 1.99
**Horse gram**	19.313^ab^ ± 1.06	22.748^bc^ ± 0.92	31.918^c^ ± 0.98	37.436^de^ ± 1.19	44.982^d^ ± 0.99
**Kabuli chickpea**	21.041^ab^ ± 1.48	26.971^abc^ ± 1.52	36.435^abc^ ± 1.43	47.669^bc^ ± 1.83	56.676^bc^ ± 2.71
**Kidney bean**	18.413^ab^ ± 0.55	21.32^c^ ± 1.52	30.18^c^ ± 1.17	35.436^e^ ± 1.13	43.835^d^ ± 0.99
**Black gram**	17.383^b^ ± 1.16	24.821^abc^ ± 1.51	33.589^bc^ ± 1.26	44.216^bcd^ ± 1.49	55.332^bc^ ± 0.81
**Green gram**	23.041^a^ ± 0.26	29.734^a^ ± 1.24	41.533^a^ ± 1.00	58.71^a^ ± 1.49	70.491^a^ ± 2.12
**Soybean**	19.186^ab^ ± 1.98	25.045^abc^ ± 1.29	35.204^abc^ ± 1.93	45.555^bc^ ± 1.75	57.421^bc^ ± 1.30
**LSD (p ≤ 0.05)**	**(5.58)**	**(5.95)**	**(6.49)**	**(7.51)**	**(8.15)**

Values represent mean of three replicates; ± values are SE; means Followed by the same letter do not differ significantly at P = 0.05 according to Tukey’s HSD test.

### Weight loss caused by pulse beetle

Weight loss displayed a trajectory comparable to grain damage (Table 5; Fig. 5). At 30 days, losses ranged from 0.54% to 2.47%, and after four months reached 17.03% (cowpea), 24.33% (chickpea), 13.68% (horse gram), 20.08% (kabuli chickpea), 11.03% (kidney bean), 18.38% (black gram), 32.88% (green gram), and 18.46% (soybean). Mean loss across the storage period varied from 1.43% to 19.61%. Across all sampling dates, losses were consistently highest in green gram and lowest in kidney bean. Overall, green gram and kidney bean differed significantly from one another and from the other hosts, representing the maximum and minimum losses, respectively. In terms of rank, green gram was followed by chickpea; kabuli chickpea, black gram, soybean, and cowpea were statistically similar to each other, and these were followed by horse gram and kidney beans.

**Table 5:** Weight loss caused by *Callosobruchus chinensis* in different pulses.

Hosts	Weight loss after indicated days of weevil release (%)				
	**30**	**60**	**80**	**100**	**120**
**Cowpea**	0.983^ cd^ ± 0.04	2.08^ cd^ ± 0.08	2.723^e^ ± 0.06	10.733f. ± 1.24	17.027^ cd^ ± 1.17
**Chickpea**	1.62^b^ ± 0.23	3.11^b^ ± 0.06	6.097^b^ ± 0.10	17.837^b^ ± 0.72	24.33^b^ ± 1.11
**Horse gram**	0.583^d^ ± 0.01	1.743^d^ ± 0.04	2.21^e^ ± 0.13	7.233^ g^ ± 0.60	13.68^de^ ± 0.61
**Kabuli chickpea**	1.89^ab^ ± 0.03	3.013^b^ ± 0.06	5.37^bc^ ± 0.21	16.907^c^ ± 0.83	20.08^bc^ ± 0.35
**Kidney bean**	0.54^d^ ± 0.06	1.19^e^ ± 0.09	2.24^e^ ± 0.14	5.063^ h^ ± 0.29	11.027^e^ ± 1.1
**Black gram**	1.527^bc^ ± 0.08	2.33^c^ ± 0.04	3.843^d^ ± 0.18	12.553^e^ ± 0.34	18.38^ cd^ ± 0.65
**Green gram**	2.473^a^ ± 0.22	4.097^a^ ± 0.16	8.46^a^ ± 0.35	21.89^a^ ± 1.28	32.88^a^ ± 1.88
**Soybean**	1.83^b^ ± 0.04	2.817^b^ ± 0.08	4.493^ cd^ ± 0.14	14.437^d^ ± 0.49	19.463^bc^ ± 0.98
**LSD (p ≤ 0.05)**	**(0.61)**	**(0.44)**	**(0.91)**	**(0.92)**	**(5.28)**

Values represent mean of three replicates; ± values are SE; means Followed by the same letter do not differ significantly at P = 0.05 according to Tukey’s HSD test.

### Effect on germination

**Fig. 6:**
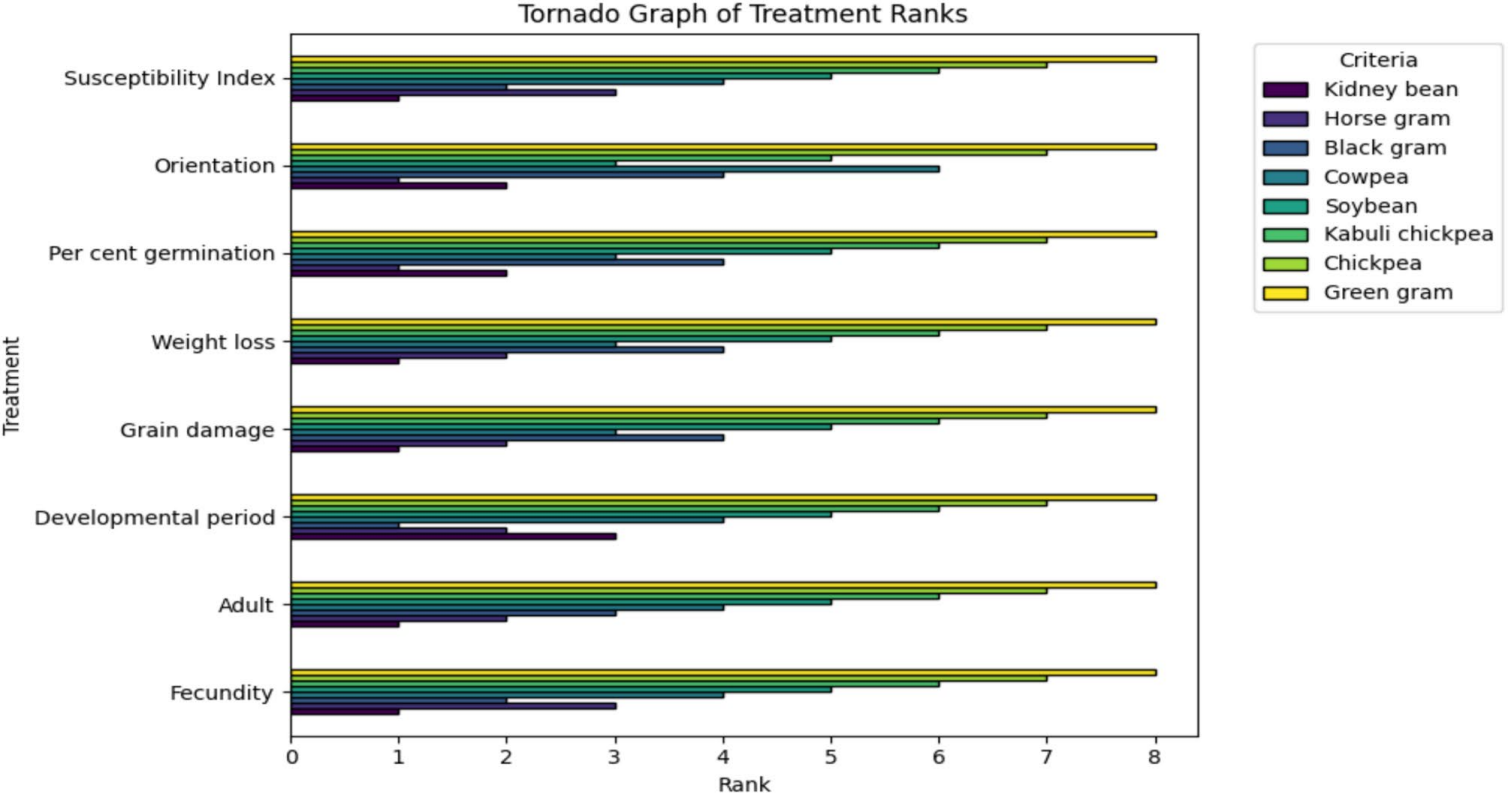
Tornado graph based on ranking of pulses in preference to *Callosobruchus chinensis*.

Seed germination assessed at 80 and 120 days post-infestation declined significantly over time in all pulses (Table 6; Fig. 6) and was inversely related to grain damage and weight loss. At 80 days, germination was highest in kidney bean (at par with horse gram) and lowest in green gram (at par with chickpea). At 120 days, germination remained highest in horse gram (at par with kidney bean) and lowest in green gram (at par with chickpea, which did not differ from kabuli chickpea).

**Table 6:** Effect of pulse beetle infestation on the germination of different pulses.

Hosts	Per cent germination after indicated days of storage	
	**80**	**120**
**Cowpea**	51.935^ab^ ± 1.23	33.583^ab^ ± 1.45
**Chickpea**	37.036^d^ ± 1.31	20.384^ cd^ ± 2.05
**Horse gram**	56.781^a^ ± 0.96	39^a^ ± 1.70
**Kabuli chickpea**	41.143^ cd^ ± 1.35	25.752^bcd^ ± 1.69
**Kidney bean**	58.595^a^ ± 2.85	38.172^a^ ± 2.75
**Black gram**	47.47^bc^ ± 1.01	27.774^bc^ ± 2.85
**Green gram**	34.415^d^ ± 1.27	16.952^d^ ± 1.78
**Soybean**	44.983^bc^ ± 1.52	27.392^bc^ ± 2.06
**LSD (p ≤ 0.05)**	**(7.56)**	**(10.26)**

Values represent mean of three replicates; ± values are SE; means Followed by the same letter do not differ significantly at P = 0.05 according to Tukey’s HSD test.

## Discussion

The findings of the current study clearly show that the pulse beetle successfully completed its life cycle and caused damage, weight loss, and reduced germination across all pulse varieties. Certain characteristics of pulses are known to make them less favorable or unsuitable for feeding, oviposition, and the development of insect pests. These factors are often influenced by biochemical properties that can alter insect behavior (antixenosis) or hinder their development (antibiosis). A single larva of *C. chinensis* can destroy several mature seeds during storage^17^. This pest is responsible for both qualitative and quantitative losses, with up to 90% damage in chickpeas^18^. The larvae infest the grains, leaving burrows filled with excrement and dead bodies, which are ground into flour or meal once processed (Atwal and Dhaliwal, 2005). It is evident from the data (Table 6) that with the increase in storage period, the per cent germination of seeds of all the pulses reduced significantly. This may be attributed to the increased levels of population build-up of the beetle and extent of damage with increase in storage period.

Host-related traits can render pulses more or less suitable for feeding, oviposition, and development in storage insects, often through biochemical factors that affect behaviour and physiology. Some studies have found little to no association between basic physical seed attributes and susceptibility to storage pests^19–21^. Comparative evidence on *C. chinensis* exhibited distinct variations in its biological performance across different host pulses, reflecting differences in host suitability. Among the tested pulses, green gram proved to be the most favourable host**,** as evidenced by the highest oviposition, shortest incubation, larval, pupal, and total development periods, highest adult emergence, longest adult longevity, and maximum susceptibility index. Varma and Anandhi^22^ showed that *C. chinensis* biology on green gram with 4.00-day incubation period, 16.40-day larval + pupal period, and adult longevity of 11.00 (male) and 9.60 (female) days, with 94% egg viability and fecundity of 85.6 eggs. Similarly, Solanki and Mittal^9^ reported that the average incubation period, larval + pupal period, and adult longevity for male and female *C. chinensis* were 4.0, 16.4, 11.0, and 9.6 days, respectively. Total developmental period was recorded as 25.2 days, with pre-oviposition, oviposition, and post-oviposition periods averaging 0.4, 8.0, and 2.2 days, respectively. Female fecundity was reported to be 85.6 eggs, with an egg viability of 94%, which were reported more similar results to our study. While Pathania and Thakur^23^ in Himachal Pradesh studied the biology of the *C. chinensis* on black gram which recorded six generations between April and October, with mean incubation, larval, pupal, and total durations of 8.00, 18.39, 8.11, and 34.50 days, longer than many of our host-specific estimates which leads to the revelling of geographic and host-dependent variability. Certain other studies on the different pulses yielded the results which are not corroborative with our results i.e. cowpea was more preferable host followed by chickpea and soybean^24–26^.

Some of the antagonistic results observed in report of Wijenayake and Karunaratne^27^ with 100% larval mortality on soybean, and faster development on cowpea whereas Bhargava et al.^28^ found fecundity ranging 47.40–75.60 eggs across seven pulses, the shortest incubation on cowpea (4.40 ± 0.54 days), and the shortest total development on cowpea (24.60 ± 1.81 days) versus the longest on soybean (44.80 ± 3.14 days). The detailed study on the susceptibility of the different pulses to *C. chinensis* is discussed based on the results observed. The choice assays revealed a robust and time-stable host hierarchy, with adults orienting most strongly to green gram at 24, 48, and 72 h, followed by consistently high responses to chickpea and cowpea, and markedly weaker attraction to kidney bean and horse gram, exact studies for the orientation of pulse beetle has not been studied but the host preference for oviposition studies has been discussed based on the reports; the widely accepted phenomenon for the host preference based on the kairomonal cues accumulate and beetles settle on grain, yielding highly significant host differences at each interval. Mechanistically, this pattern is concordant with known drivers of bruchid host use: attractive volatile blends from green gram that guide host location; favourable seed traits (e.g., thinner, smoother coats, higher available nutrients and moisture) that promote oviposition and early larval success on green gram and chickpea; and deterrent or mechanical barriers (e.g., thicker, harder coats, surface wax chemistry, and higher phenolics) that reduce acceptance and retention on kidney bean and horse gram^29–31^. The concordance between orientation rank and recognized performance/susceptibility gradients strengthens the inference that adult orientation is a reliable early predictor of downstream infestation pressure, with green gram at greatest risk across the observation window and kidney bean/horse gram relatively less susceptible under comparable storage conditions.

The percent grain damage by *C. chinensis* increased progressively with storage duration in all tested pulses. Such cumulative losses are typical of bruchid infestations in sealed or bulk storage systems, where overlapping generations amplify population density over time (Ali et al., 2020)^32^. The 4.8–5.8-fold increase in grain damage from 30 to 120 days observed here is comparable with earlier findings where storage losses in susceptible pulses reached 30–100% under ambient conditions if unmanaged^33,34^. Among the host species, green gram suffered the greatest damage, significantly exceeding all other pulses. This aligns with earlier reports that green gram and chickpea are highly suitable hosts for *C. chinensis*, owing to shorter developmental periods, higher emergence rates, and longer adult longevity on these hosts^35,36^. In contrast, kidney bean consistently showed the least damage, followed by horse gram, both of which are recognized as poor hosts due to harder seed coats and unfavourable biochemical profiles that deter oviposition and larval penetration^37^.

Interestingly, moderate damage was recorded on soybean in the present study, whereas several authors have reported little or no adult emergence on soybean^38^. Such variability may reflect genotypic differences in seed-coat composition and anti-nutritional factors, suggesting that host susceptibility within species may not be uniform^37,38^. The relatively high susceptibility of kabuli chickpea compared with desi chickpea observed here also supports the role of seed morphology and nutrient content in determining damage levels. Overall, *C. chinensis* is capable of infesting a wide range of pulses, susceptibility is strongly host-dependent. Green gram, chickpea, and kabuli chickpea require particular attention in storage protection, as they are more prone to rapid deterioration, whereas kidney bean and horse gram can be stored with relatively less risk.

This study revealed that weight loss exhibited a progressive trend comparable to grain damage across all host pulses, reaffirming the reliability of both parameters as indicators of *C. chinensis* impact. At 30 days post-infestation, weight loss was minimal (0.54–2.47%), but losses increased sharply with storage duration, reaching up to 32.88% in green gram after 120 days. Significant variation among hosts was evident, with green gram consistently showing the highest loss and kidney bean the least, indicating differential host suitability. These findings corroborate earlier reports that mung bean is highly susceptible to *C. chinensis*^39,40^, while kidney bean is an unsuitable host due to poor oviposition and limited larval development (Rajapakse, 2006, Islam et al., 2014). Intermediate levels of weight loss observed in chickpea, kabuli chickpea, black gram, soybean, and cowpea suggest moderate susceptibility, whereas horse gram and kidney bean consistently experienced the least damage. Collectively, the results confirm that weight loss, in conjunction with grain damage, provides a robust measure of host susceptibility, highlighting green gram as the most vulnerable pulse and kidney bean as the most resistant under bruchid infestation.

The decline in seed germination observed at 80 and 120 days post-infestation was closely associated with the extent of grain damage and weight loss, reaffirming the destructive impact of *C. chinensis* on both seed quality and viability. Green gram, which recorded the highest levels of grain damage and weight loss, also exhibited the lowest germination percentages, indicating that the severe structural and physiological damage caused by larval feeding compromises embryo viability. Similar observations have been reported by^39–41^, who showed that bruchid infestation, not only reduces seed mass but also severely impairs viability. The reduction in germination is attributed to direct consumption of the cotyledons and embryo^17,42^ and increased fungal invasion facilitated by seed perforations^43^.

In contrast, kidney bean and horse gram consistently showed the lowest grain damage and weight loss, and accordingly maintained higher germination percentages across storage intervals. This suggests that the inherent resistance of these hosts restricts bruchid development, possibly due to physical traits such as seed coat hardness and chemical deterrents^44^ (Appleby & Credland, 2003). Intermediate germination losses recorded in chickpea, kabuli chickpea, black gram, soybean, and cowpea paralleled their moderate levels of damage and weight reduction, further supporting the direct relationship between infestation intensity and seed viability^45,46^. The present findings emphasize that seed germination is a sensitive indicator of bruchid-induced deterioration. Pulses such as green gram, being highly susceptible, are unsuitable for long-term storage, while kidney bean and horse gram offer greater stability under storage conditions, in agreement with earlier reports by^47–49^. The ranking of different pulses were prepared in the present investigation, according to the foresaid parameters (Table 7, Fig. 3).

**Table 7:** Ranking of pulses in order of relative preference to *Callosobruchus chinensis*.

Pulses	Ranks based on different parameters								Overall rank	Rank* position
	**Fecundity**	**Adult** **emergence**	**Developmental period**	**Grain damage**	**Weight loss**	**Per cent germination**	**Orientation**	**Susceptibility Index**		
**Kidney bean**	1	1	3	1	1	2	2	1	12	**1**
**Horse gram**	3	2	2	2	2	1	1	3	16	**2**
**Black gram**	2	3	1	4	4	4	4	2	24	**3**
**Cowpea**	4	4	4	3	3	3	6	4	31	**4**
**Soybean**	5	5	5	5	5	5	3	5	38	**5**
**Kabuli chickpea**	6	6	6	6	6	6	5	6	47	**6**
**Chickpea**	7	7	7	7	7	7	7	7	56	**7**
**Green gram**	8	8	8	8	8	8	8	8	64	**8**

*Rank (1 to 8): 1—least preferred8–- Most preferred.

Several factors influence the oviposition behavior of *C. chinensis*, including seed size, surface smoothness, seed coat depth, and color, which are all important for egg-laying. The temperature and humidity also significantly impact oviposition behavior, with egg numbers highest on the first day and decreasing over time^11^. A thicker seed coat may hinder development by preventing larvae from entering the seed and adults from emerging. Additionally, phenol content in seeds may extend the developmental period by inhibiting the pest’s development^50^. However^51^, found that characteristics like seed size, shape, color, volume, and seed coat texture did not significantly influence the oviposition preference of *C. chinensis* in stored mung beans^52^ studied various pulse genotypes and found that chickpea had the highest number of eggs deposited (73.1), while black gram had the lowest (19.5). Chickpea also experienced the highest seed weight loss (19.9%), while black gram had the lowest (7.6%). Based on seed weight loss, black gram and mung bean were classified as tolerant, lentil as moderately susceptible, and chickpea as susceptible. A positive correlation was observed between weight loss and egg deposition in all genotypes. In contrast, in our study, green gram, chickpea, and cowpea were the most susceptible, while kidney bean and horse gram were somewhat tolerant^8^ carried out surveys in eight different locations of Himachal Pradesh to assess the damage caused by *C. chinensis*. They reported damage and weight loss of grains in all eight districts which included green gram, black gram, kidney bean, chickpea, soybean and cowpea.

Mofunanya and Namgbe (2016) found significant reductions in moisture, protein, and carbohydrate levels in infested *V. unguiculata* seeds, while ash, fiber, and fat content increased. Infestation also raised the concentrations of minerals such as Zn, Mn, K, Ca, Na, and Co, but decreased levels of Ni, Fe, Cu, and Mg. Additionally, infestation led to a reduction in vitamin B and A content, while increasing the levels of vitamins E and C. According to Chakraborty and Mondal^53^, various morphological traits, including texture, seed size, weight, volume, and colour, influence bruchid oviposition preferences among different pulses. They indicated that in a free choice setting, bruchids favour dark and brown seeds over white ones for laying eggs. The smoothness of the seed surface, thickness of the seed coat, and chemical cues also impact the oviposition and damage by the pulse beetle (*C. chinensis*) on various pulse genotypes, including lentils, mung beans, chickpeas, and black gram (Salim et al*.*, 2018).^54^ reported that greater seed weight and a thick seed coat prolong various developmental stages of *C. chinensis*. Despite the increased nutritional content, the development is hindered due to the thick seed coat obstructing the entry of young larvae into the seed and the emergence of adults from within it. There are reports that phenol likely plays a role in inhibiting or interfering with the development process of the pulse beetle^53^. Probably the higher phenol content also extends the developmental period of *C. chinensis*^55^.

Further studies incorporating additional physiological, biochemical, or ecological parameters may provide deeper insights into host-mediated variation in the development and reproductive potential of *C. chinensis.*

## Conclusions

This study highlights the critical role of host-related traits in governing the susceptibility of pulses to *Callosobruchus chinensis*. Based on our findings, it can be concluded that the pulse beetle successfully completed its life cycle on all the pulses tested. However, there were notable differences in the biological parameters of the beetle across the pulses. Green gram consistently exhibited the highest vulnerability, sustaining rapid population growth of the pest, extensive grain damage, severe weight loss, and the lowest germination rates. In contrast, kidney bean and horse gram displayed relative resistance, with restricted bruchid development and minimal storage losses, while chickpea, kabuli chickpea, black gram, soybean, and cowpea showed moderate susceptibility. These findings reaffirm that grain damage, weight loss, and seed viability are reliable indicators of host suitability for bruchid infestation. These differences in losses may be linked to various physical characteristics of the seeds, such as color, thickness, size, and moisture content, as well as biochemical factors like carbohydrate, protein, fat, phenol, and vitamin content. Although the pulses in this study were not analyzed for their biochemical composition, further research on these parameters could help clarify the relationship between seed characteristics and the beetle’s biological response.

From a management perspective, the results underscore the need for host-specific storage strategies, with particular attention to highly susceptible pulses such as green gram and chickpea that require early protection through improved storage structures, prophylactic treatments, or integration of microbial and botanical protectants. Moreover, the consistent resistance observed in kidney bean and horse gram highlights their potential as donor parents in breeding programs aimed at developing bruchid-resistant varieties. The study also reinforces the importance of linking host biology with pest management, providing a foundation for sustainable strategies to minimize post-harvest losses and ensure seed quality during storage.

## Data Availability

The datasets used and/or analysed during the current study available from the corresponding author on reasonable request.
